# Triaxial Mechanical Properties and Mechanism of Waterborne Polyurethane-Reinforced Road Demolition Waste as Road Bases

**DOI:** 10.3390/polym14132725

**Published:** 2022-07-03

**Authors:** Beifeng Lv, Yinuo Zhao, Na Li, Yanfei Yu, Yanting Wu, Miaojie Gu

**Affiliations:** School of Civil Engineering, Shaoxing University, Shaoxing 312000, China; 20020852047@usx.edu.cn (B.L.); cosm1cc@163.com (Y.Z.); yuyanfei@usx.edu.cn (Y.Y.); 19020852065@usx.edu.cn (Y.W.); gmjzoe@163.com (M.G.)

**Keywords:** recycled aggregate, waterborne polyurethane, mechanical properties, reinforcement mechanism

## Abstract

The recycling and reuse of construction waste have not only effectively protected natural resources but also promoted the sustainable development of the environment. Therefore, in this article, waterborne polyurethane (WPU) as a promising new polymer reinforcement material was proposed to reinforce the road demolition waste (RDW), and the mechanical performance of WPU-reinforced RDW (named PURD) was investigated using triaxial unconsolidated and undrained shear (UU) and Scanning Electron Microscope (SEM) tests. The results showed that under the same curing time and confining pressure, the shear strength of PURD increased with the increase in WPU content. When the WPU content was 6%, the WPU presented the best reinforcement effect on RA. The failure strain of PURD increased with the increase in confining pressure, but increased first and then reduced with the increase in WPU content. The specimens with 5% WPU content showed the best ductility. At the curing time of 7 and 28 days, the internal friction angle and cohesion of PURD increased with the increase in WPU content, and they reached a maximum when the WPU content was 6%. The internal friction angle barely budged, but the cohesion increased obviously. The enhancement effect of WPU was attributed to the spatial reticular membrane structure produced by wrapping and bonding particles with the WPU film. Microscopic analysis showed that with the increase in WPU content, the internal pore and crack size of PURD gradually decreased. As the WPU content increased, the WPU film became increasingly thicker, which increased the adhesion between WPU and RA particles and made the structure of PURD become increasingly denser.

## 1. Introduction

Over the past few decades, infrastructure construction has flourished due to widespread urbanization, the world population soaring, and the changing economic landscape of the developing countries [1,2]. This has led to a sharp increase in the amount of demolition waste from old buildings, thereby posing a serious burden on the ecological environment and human health [3,4,5]. Additionally, due to the construction of a large number of new construction projects, the serious shortage of natural resources and the extensive mining methods have destroyed the ecological balance of the production area [6,7,8,9,10]. The world has been persistently striving to create a development environment with steady progress in material, energy, production, economy, environment, and efficiency. The construction industry can achieve this by effectively reusing demolition waste as building materials [11,12]. Therefore, the construction demolition waste is used to replace natural building materials in current society, which will make great contributions to the sustainable development [13,14,15].

In the process of pursuing sustainable development in the construction industry, cement production consumes a lot of energy and emits a large amount of greenhouse gases, and the total emission accounts for about 8% of the total global carbon dioxide emissions [16,17,18]. The high-molecular polymers present fast hardening, micro-expansion, stable strength, and good mechanical properties, and they can be used to replace cement materials and widely used in infrastructure construction fields such as road construction, slope stabilization, foundation, and roadbed treatment [19,20,21]. Polyurethane as a high-molecular polymer is formed by the reaction of diisocyanate and polyol (or equivalent) in the presence of a catalyst. Polyurethane has good biocompatibility, hydrolysis stability, and chemical resistance [22,23,24]. According to the preparation process, polyurethane can be divided into solvent and waterborne. Compared with traditional solvent polyurethane, waterborne polyurethane (WPU) is more environmentally friendly, and there is a great demand in the fields of coatings, thickeners, sealants, and adhesives [25,26].

Currently, the high-molecular polymers have been applied in the reinforcement of building materials, among which polyurethane is particularly prominent in improving strength and stability [27,28,29,30]. Wei et al. [31] conducted a series of unconfined compressive strength tests and scanning electron microscope tests with different WPU contents to explore the reinforcement effect of WPU on sand, showing that with the increase in WPU content, the unconfined compressive strength and residual strength of the sand increase, and the strength and ductility of sand can be effectively improved. Because WPU has a unique reticular membrane structure, the WPU can improve the cohesion between sand particles. In order to study the influence of polyurethane on the permeability of sand, Liu et al. [32] carried out a series of reinforcement layer form tests, single-hole permeability tests, and porous permeability tests on the sand reinforced by polyurethane. The test results showed that the impermeability of the sand reinforced by polyurethane is improved. With the increase in polyurethane concentration, the thickness and complete degree of the reinforcement layer increase, and the permeability coefficient decreases. Zhang et al. [33] studied the effect of polyurethane content on the mechanical properties and microstructure of cement mortar, with the results showing that with the increase in polyurethane content, the compressive strength of cement mortar gradually decreases but the flexural strength gradually increases. The polyurethane helps to optimize the microstructure of cement mortar and inhibit crack propagation. Samaila et al. [34] investigated the marine clay treated with polyurethane (PU); it was found that the compressive strength of marine clay specimens is effectively improved; and the failure strain, compression index, and expansion index are reduced by PU. The microstructure of marine clay specimens treated with PU becomes more compact. Liu et al. [35] used cyclic triaxial tests to investigate the effect of PU on the dynamic characteristics of crushed stone, and in this work, the effects of confining pressure, PU content, consolidation stress ratio, and loading frequency on the shear modulus and damping ratio of polyurethane-reinforced crushed stone were considered. The results showed that increasing the confining pressure, polyurethane content, and consolidation stress ratio can increase the maximum shear modulus and reduce the damping ratio of PU-reinforced crushed stone. However, the shear modulus of PU-reinforced crushed stone slightly increases with the increase in loading frequency, but the damping ratio is not sensitive to it. In order to explore the effect of waterborne polyurethane on the mechanical properties, durability, and microstructure of concrete, Fan et al. [36] carried out a series of experimental research and theoretical analysis. The test results showed that the compressive strength, splitting tensile strength, flexural strength, and elastic modulus of concrete can be improved by adding an appropriate amount of waterborne polyurethane. Meanwhile, under the action of waterborne polyurethane, the chloride penetration resistance, impermeability, freezing and thawing resistance, and microstructure of concrete have also been significantly improved.

In summary, it is found that due to the pollution problems caused by the use of cement and the accumulation of construction waste, as well as the shortcomings of high energy consumption in the mining of construction materials, the current society urgently needs to find new environmental protection reinforcement materials and resource utilization methods of construction waste to alleviate the pressure on the environment and resources. At present, a large number of research works mainly focuses on natural building materials such as sand, marine clay, gravel, and concrete, while the research on recycled building materials is relatively rare. Therefore, using waterborne polyurethane as a new environmental protection reinforcing material to study its effect on the mechanical properties of recycled aggregate can realize the engineering recycling of building materials, to achieve the social benefits of protecting the environment and saving resources. Meanwhile, the abundant research work has only explored the compressive strength, flexural strength, shear modulus, and damping ratio of various building materials, and has not yet conducted in-depth research on shear strength.

The objective of this paper was to explore the effect of WPU on the triaxial shear strength of RA by unconsolidated undrained shear (UU) tests on the WPU-reinforced RA (PURD) specimens under different ages, confining pressures, and contents of waterborne polyurethane. Meanwhile, the microstructure of PURD was characterized by scanning electron microscope (SEM) tests to explore the WPU reinforcement mechanism on RA.

## 2. Experimental Materials, Schemes, and Methods

### 2.1. Materials

**Figure 1:**
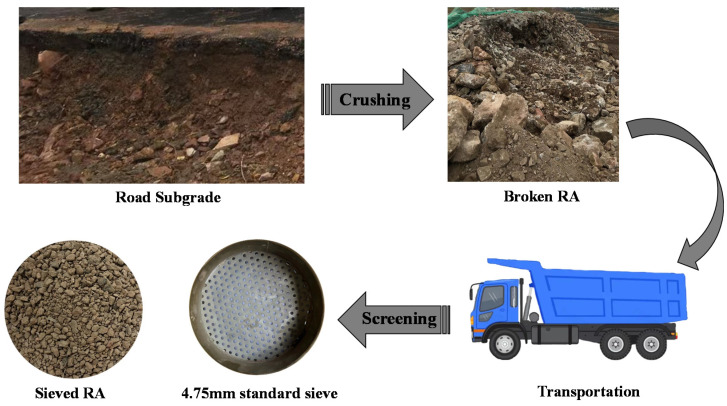
Acquisition process of RA required for test.

**Figure 2:**
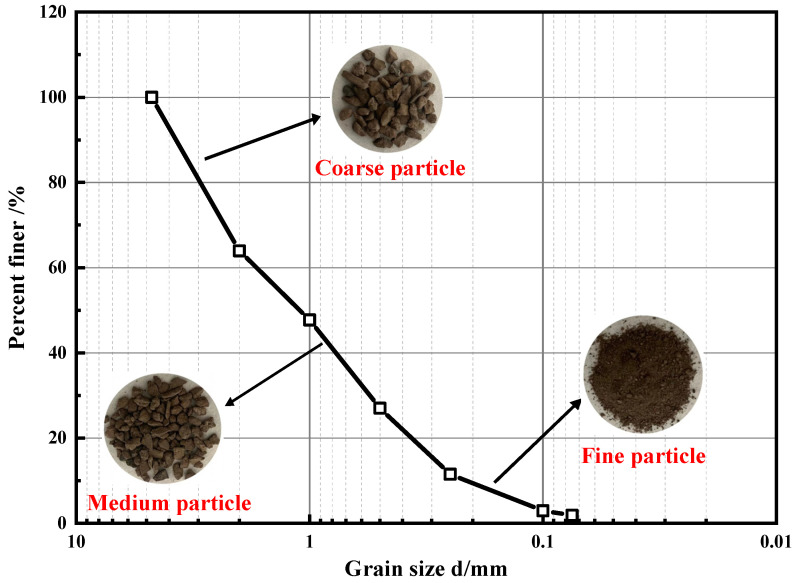
Particle grading curve.

The RA was collected from the abandoned road section of Erhuan North Road, Shaoxing City, Zhejiang Province, which was the mixed gravel after the crushing of the roadbed base material. The main chemical components of RA are SiO_2_ and CaCO_3_. The acquisition process of RA required for the test is shown in Figure 1. According to the Test Methods of Materials Stabilized with Inorganic Binders for Highway Engineering (JTG E51-2009) [37], fine-grained gravel soil with a particle size of less than 4.75 mm was selected as the RA used in the test, and the particle grading curve is shown in Figure 2. According to the Test Methods of Soils for Highway Engineering (JTG 3430-2020) [38], the physical performance indexes of RA were tested, and the results are shown in Table 1. The WPU is produced by Shenzhen, China, Jitian Chemical Co., Ltd. The model is F0410 and its main performance indexes are shown in Table 2.

### 2.2. Experimental Scheme

The RA was reinforced by WPU with four contents. The WPU solutions were diluted with water to a solution with a low content, and then the RA was mixed with WPU. The specific test scheme is shown in Table 3. For the convenience of expression, PURD-X is used to represent the specimen number, where X represents the content of WPU (%). Meanwhile, the SEM tests were performed to reveal the strengthening mechanism of mechanical properties of WPU-reinforced RA.

The WPU content in the test scheme is the mass ratio of WPU and RA, and the solid content of the solution needs to be considered. The moisture content of the specimen was set to 16%, the main purpose was to keep consistent with the natural moisture content of the RA, and the moisture contained in the WPU solution should be considered. The calculation process of the test mix proportion is shown in Equations (1) and (2).

(1) $$m_{\mathrm{WPU}}=\frac{m_{\mathrm{RA}}\times r_{\mathrm{WPU}}}{40\%}$$

(2) $$m_{w}=(m_{\mathrm{RA}}+m_{\mathrm{WPU}}\times 40\%)\times 16\%-m_{\mathrm{WPU}}\times \left(1-40\%\right)$$

where $m_{\mathrm{WPU}}$ is the mass of the WPU solution (g), $r_{\mathrm{WPU}}$ is the content of the WPU (%), $m_{\mathrm{RA}}$ is the mass of RA (g), and $m_{w}$ is the mass of water (g).

### 2.3. Specimen Preparation

**Figure 3:**
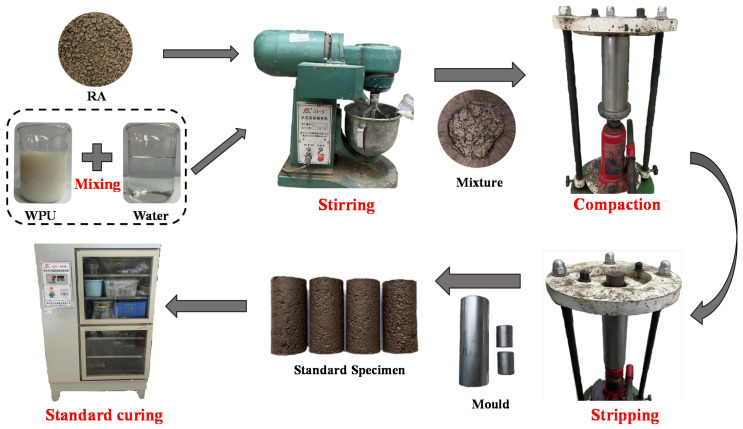
Specimen preparation process.

The preparation process of the UU test specimens in this study is divided into the following steps (see Figure 3) as per the Standard for geotechnical testing method (GB/T50123-2019) [39] and the designed experimental scheme.

(1) The RA was placed in a constant-temperature oven at 105 °C for 24 h to ensure that the moisture in the RA was completely dried, and then cooled to room temperature.
(2) The corresponding qualities of the RA, WPU solution, and water were weighed according to the mix proportion set in Table 3. Then, the weighed water was slowly poured into the WPU solution while stirring. Afterward, the WPU solution was mixed with the RA.
(3) An amount of 154 g of the mixture stirred evenly was shaped in a cylindrical mold with a height of 80 mm and a diameter of 39.1 mm. It must be fully compacted with a jack and statically pressed for 10 min.
(4) The specimens were demolded after being fully compacted. Then, the specimens were cured in a standard curing box for 7 and 28 days, respectively. The curing temperature was controlled at 20 ± 2 °C, and the relative humidity was >95%.

The samples for the SEM test were from the dried sample blocks of broken sample in the UU test. A layer of conductive adhesive was pasted tightly on the sample with a thin rod, and then the specimen particle powder on the surface was blown away with a suction balloon to avoid polluting the instrument lens.

### 2.4. Experimental Method

#### 2.4.1. UU Test

The instrument used in the UU test was a fully automatic triaxial shearing instrument (TKA.TTS.3S) produced by Nanjing, China, TKA Technology Co., Ltd. The four confining pressures (100, 200, 300, and 400 kPa) were applied, and the loading rate was set to 1 mm/min. According to the relevant provisions of the Standard for geotechnical testing method (GB/T 50123-2019) [39], after the deviatoric stress reached the maximum, the axial strain increased by 3–5% and the test was stopped. In this article, the test was terminated when the axial strain reached 14%.

#### 2.4.2. SEM Test

The equipment used for the SEM test was a high- and low-vacuum scanning electron microscope (JSM-6360LV), which is produced by Tokyo, Japan, Electronics Co., Ltd. The specimens damaged from the UU test were placed in an oven for 24 h, and the test was performed after the specimen was dry. Finally, the SEM images were taken under different magnifications, and the magnifications of this study were selected as 500× and 2000×.

## 3. Results and Discussions

### 3.1. Deviatoric Stress–Strain Behaviors

**Figure 4:**
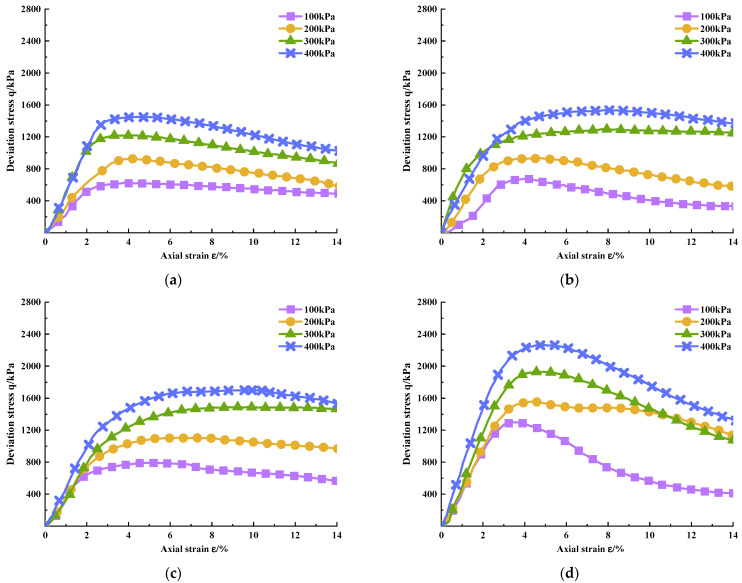
Deviatoric stress–strain curves of PURD at 7 days of curing time. (**a**) PURD-3; (**b**) PURD-4; (**c**) PURD-5; (**d**) PURD-6.

**Figure 5:**
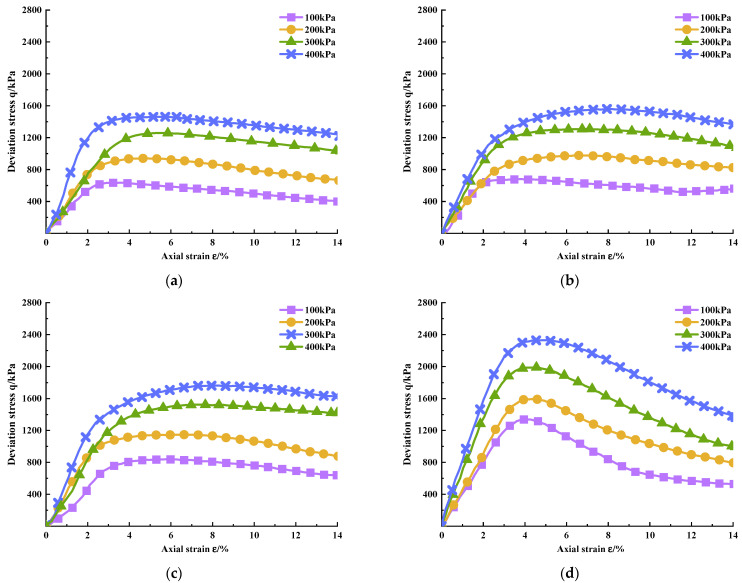
Deviatoric stress–strain curves of PURD at 28 days curing time. (**a**) PURD-3; (**b**) PURD-4; (**c**) PURD-5; (**d**) PURD-6.

According to the deviatoric stress q and the axial strain ε obtained from the test, the deviatoric stress–strain relationship curves of PURD specimens are drawn in Figure 4 and Figure 5 at 7 and 28 days of curing time, respectively. From Figure 4 and Figure 5, the deviatoric stress–strain curves of PURD at 7 and 28 days of curing time presented strain softening behaviors, namely, the deviatoric stress first increased and then decreased with the increase in strain. The softening characteristics of PURD-6 became more obvious than those of PURD-3, PURD-4, and PURD-5. The deviatoric stress of PURD-6 had an obvious decreasing trend after reaching the peak stress, and the strain softening behavior became more obvious especially under a lower confining pressure.

**Figure 6:**
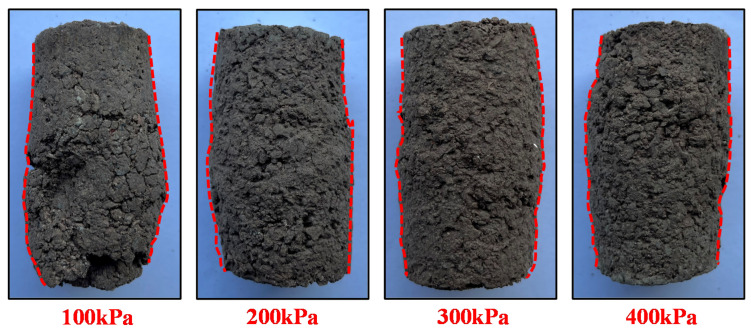
Failure mode of specimens.

Figure 6 shows the specimen failure modes of PURD specimens under different confining pressures. From Figure 6, the failure form of the specimens was bulging failure under different confining pressures. As the confining pressure increased, the bulging of the specimens became more obvious. When the confining pressure was 100 kPa, bulging cracks appeared on the surface of the specimen. First, this is mainly because the bonding and cementation effect of WPU can effectively inhibit the sliding of RA particles in the specimen to a certain extent [40]. However, under low confining pressure, the internal structure of the specimen has poor resistance to external loads. Under the action of external load, the friction and bite force between RA particles cannot inhibit its deformation, which will lead to the failure of the specimen [41]. Under high confining pressure, the specimen is constrained by large peripheral pressure, which makes the RA particles in the specimen subject to a certain binding effect, so it is not easy to slide and cause specimen damage [42,43].

### 3.2. Shear Strength

**Figure 7:**
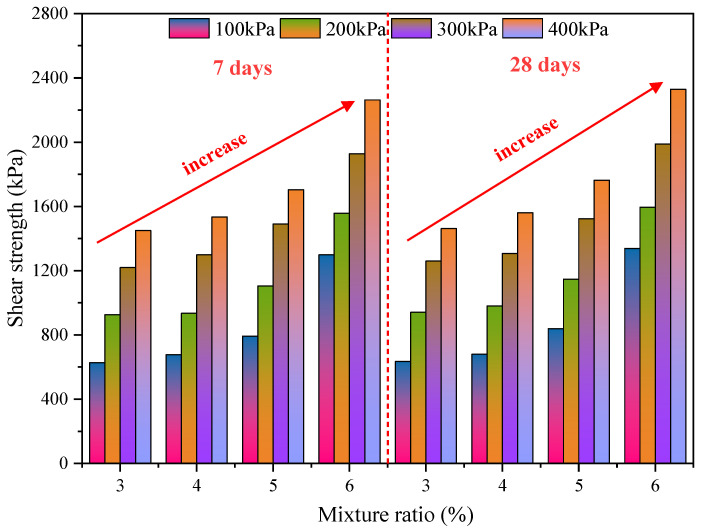
Shear strength.

According to the deviatoric stress–strain curves of PURD from Figure 5, the peak deviatoric stress under different confining pressures can be obtained, which is defined as the peak strength *q*_max_, kPa, which can reflect the shear strength of PURD. Figure 7 shows the variation law of shear strength of PURD at different curing times. From Figure 7, it is found that the peak strength of PURD with different WPU contents increased gradually with the increase in confining pressure. Meanwhile, it can be found that the change trend of peak strength curves of PURD-3, PURD-4, PURD-5, and PURD-6 at 7 and 28 days was basically the same. Therefore, the PURD with different WPU contents had the same variation law of peak strength.

By further analyzing Figure 7, the variation law of the peak strength of PURD with WPU content is obtained. Under the same curing time and the same confining pressure, the peak strength of PURD increases with the increase in WPU content. Meanwhile, the increase range of peak strength becomes increasingly more obvious with the increase in WPU content. At the curing time of 7 and 28 days, under the same confining pressure, the peak strength of PURD-4 increases by less than 10% compared with that of PURD-3. The peak strength of PURD-5 increases by 10% to 25% compared with that of PURD-4. The peak strength of PURD-6 increases by 30% compared with that of PURD-5, and when the confining pressure is 100 kPa, the rising range reaches 64%. Therefore, a 6% content of WPU can make PURD have the highest shear strength and the best reinforcement effect on RA. The important effect of WPU content on the shear strength of PURD is mainly because with the increase in WPU content, the polyurethane itself will produce a higher bonding effect and participate in more curing reactions, which will affect the RA. The bonding between particles becomes increasingly stronger, which makes the internal connection of PURD closer, so the shear strength of WPU will gradually increase with the increase in WPU content.

The curing time of cement-based materials has an important influence on the strength of the specimen. Cement usually completes almost all hydration reactions when curing for 28 days, and the strength of the specimen will increase significantly compared with that under the curing time of 7 days. Therefore, it is necessary to explore the effect of curing time on WPU-strengthening RA [44,45]. Compared with Figure 7, the peak strength of PURD with each content at the curing time of 28 days increases slightly compared with 7 days, and the increase range is less than 6%, which is almost negligible. It can be concluded that the curing time has little effect on WPU strengthening RA, and also has little effect on the shear strength. The above analysis shows that WPU can directly exert all its strength effects in a short period of time, and does not require a long curing period. Therefore, WPU has relatively low requirements on construction time and is relatively flexible in engineering applications, which is a very effective and promising new type of high-molecular polymer reinforcement material.

### 3.3. Failure Strain

**Figure 8:**
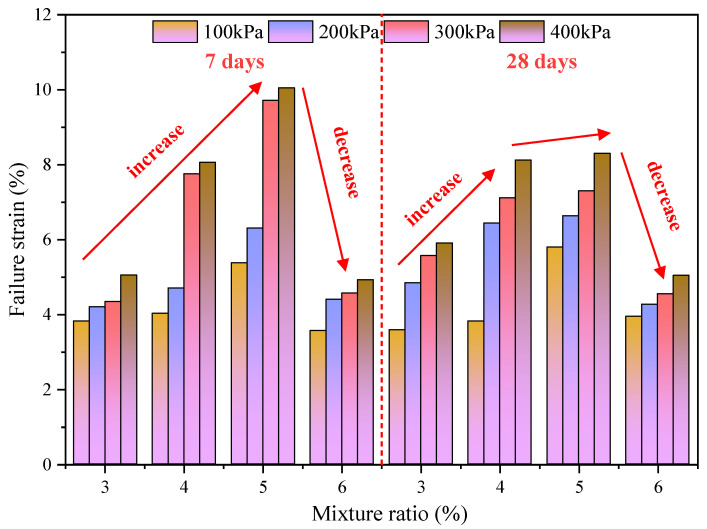
Failure strain.

The strain corresponding to the peak strength of PURD in the deviatoric stress–strain curve in Figure 5 and Figure 6 is called the failure strain [46], and Figure 8 shows the change law of failure strain of the specimens. The failure strain can describe the ductility of the specimen to a certain extent. The larger the failure strain value is, the later the shear failure of the specimen occurs [47,48].

According to Figure 8, the failure strain of PURD with different WPU contents increases with the increase in confining pressure at 7 and 28 days of curing time. On the whole, the change range of the failure strain of PURD relative to the confining pressure is relatively gentle. Only the failure strains of PURD-4 and PURD-5 show a significant increase between 100 kPa to 200 kPa and 200 kPa to 300 kPa confining pressures. However, the effect of curing time on the failure strain of PURD does not show a particularly obvious rule, and the overall difference is not significant.

Comparing the failure strain of PURD with different WPU contents, it can be found that the failure strain of PURD increases first and then decreases with the increase in WPU content under the curing time of 7 and 28 days. Under two curing times and four confining pressures, the failure strain of PURD-5 reaches the maximum value. Compared with PURD-3, the failure strain increases by 41%, 50%, 124%, and 99% at the curing time of 7 days, and increases by 61%, 37%, 31%, and 41% at the curing time of 28 days, respectively. The growth rate is relatively large. The above analysis shows that PURD-5 exhibits the best ductility, mainly because the increase in WPU content will make the cementation between the RA particles more obvious, and there will be a certain “pulling” effect between particles to prevent brittle failure [49]. However, when the content of WPU is too much, a large number of agglomerates will appear in the PURD, and the dispersion is not uniform, which inhibits the ductility of the specimen.

### 3.4. Shear Strength Parameters

**Figure 9:**
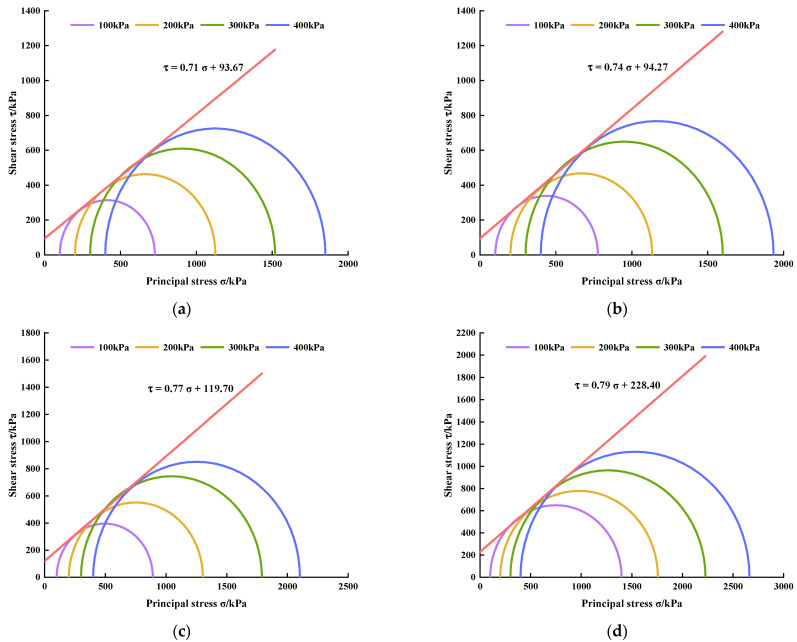
Limit Mohr stress circles and shear strength envelopes of PURD at 7 days of curing time. (**a**) PURD-3; (**b**) PURD-4; (**c**) PURD-5; (**d**) PURD-6.

**Figure 10:**
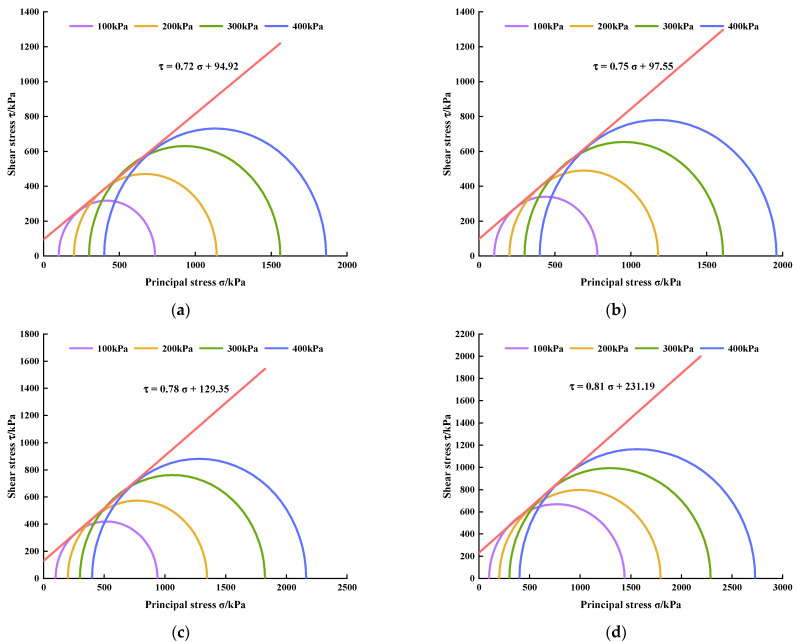
Limit Mohr stress circles and shear strength envelopes of PURD at 28 days of curing time. (**a**) PURD-3; (**b**) PURD-4; (**c**) PURD-5; (**d**) PURD-6.

Taking the normal stress *σ* as the abscissa, the shear stress *τ* as the ordinate, (*σ*_1f_ + *σ*_3f_)/2 as the center, and (*σ*_1f_ − *σ*_3f_)/2 as the radius, the limit Mohr stress circle is drawn on the *τ*-*σ* stress plane diagram. Then, the common tangents of the Mohr circle under four different confining pressures are drawn, which are the shear strength envelopes of the limit Mohr stress circle, where *σ*_1_ represents the large principal stress, *σ*_3_ represents the small principal stress, and the subscript *f* represents the limit equilibrium state, that is, the molar stress circle is tangent to the shear strength envelope, which means that the shear stress on the *τ*-*σ* stress plane is equal to the shear strength. Based on this, the limit Mohr stress circles and shear strength envelopes of PURD with different WPU contents under 7 and 28 days of curing time were drawn, respectively (see Figure 9 and Figure 10).

**Figure 11:**
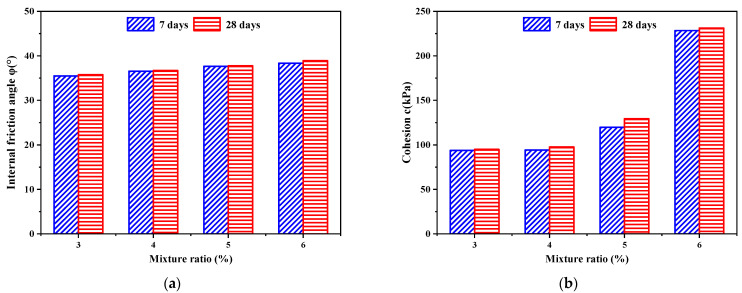
Shear strength parameters. (**a**) Internal friction angle *φ*; (**b**) cohesion *c*.

Figure 11 shows the variation law of the internal friction angle *φ* and cohesion *c* of PURD specimens obtained from the shear strength envelopes. As can be seen from Figure 11, at the curing time of 7 and 28 days, the internal friction angle and cohesion of PURD show an increasing trend with the increase in WPU content and reaches the maximum when the WPU content is 6%. The change range of internal friction angle is relatively small, with an increase of only about 5%, while the increase in cohesion is more obvious. Under 7 days of curing time, the cohesion of PURD-6 increases by 144%, 142%, and 91% compared with those of PURD-3, PURD-4, and PURD-5, respectively. Under 28 days of curing time, the cohesion of PURD-6 increases by 141%, 134%, and 77% compared with those of PURD-3, PURD-4, and PURD-5, respectively. Meanwhile, compared with the test results in Section 2.2, it can be found that the variation rules of the internal friction angle and cohesion of PURD are highly consistent with the variation rules of the shear strength. However, the content of WPU has a limited effect on the internal friction angle of PURD. As a polymer curing time, WPU has a high degree of fluidity, and its consolidation effect cannot significantly change the internal friction angle of RA. Therefore, it can be known that WPU mainly improves the shear strength by increasing the cohesion of the specimen.

Under the same WPU content, the internal friction angle and cohesion of PURD both increase with curing time, but not obviously. The strength of PURD is mainly provided by the bite force and friction generated by the mutual extrusion of RA particles and the bonding force generated by the curing reaction of WPU. The increase in internal friction is mainly due to the curing reaction between WPU and water in the specimen, and the curing reaction will gradually end with the increase in curing time, resulting in the decrease in water in the specimen. The internal friction angle will increase with the decrease in water content, and the decrease in water content will lead to the increase in bite force and friction between RA particles [50]. Additionally, WPU itself is also prone to oxidative consolidation reaction, which will produce high-strength adhesion and can better bond loose RA particles together. Moreover, a large number of long-chain macromolecules and isocyanate groups in WPU make it have a special polymer reticular structure, which can significantly improve the cohesion of the specimen [31,32]. With the increase in curing time, the consolidation reaction between WPU and water and the oxidative consolidation reaction of WPU will proceed more completely, thereby further increasing the cohesion of PURD.

## 4. Mechanism Analysis

**Figure 12:**
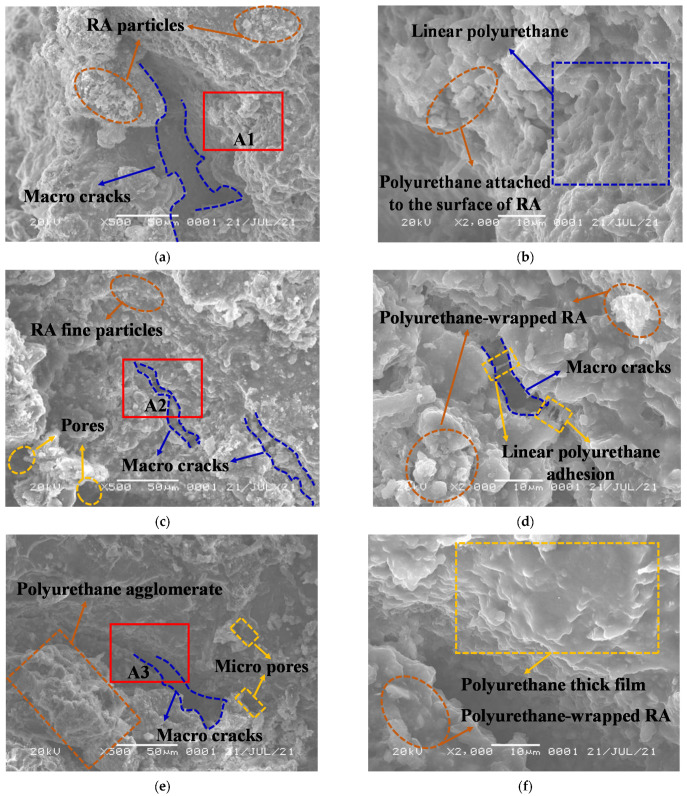

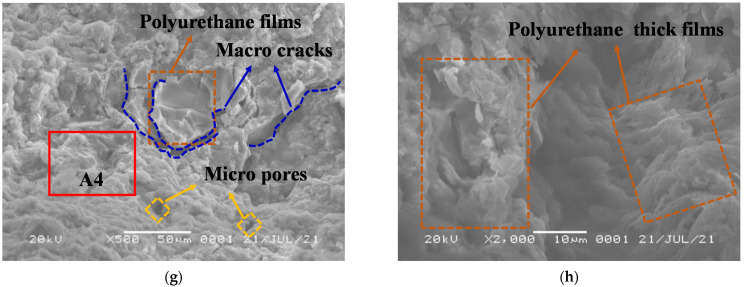
SEM images of PURD with different WPU contents. (**a**) PURD-3, 500 times; (**b**) PURD-3, 2000 times; (**c**) PURD-4, 500 times; (**d**) PURD-4, 2000 times; (**e**) PURD-5, 500 times; (**f**) PURD-5, 2000 times; (**g**) PURD-6, 500 times; (**h**) PURD-6, 2000 times.

Through the above UU test results and analysis, it has been found that WPU has an important effect on the mechanical properties of RA, and the variation law of the shear strength of PURD with different WPU contents has been obtained. However, the above results are analyzed from the macro-mechanical level, which has the disadvantage of relative superficiality. Therefore, we can try to make an in-depth analysis of the mechanism of WPU enhancing RA from the micro-level. Therefore, the SEM test was performed on PURD with different WPU contents, and the SEM images at 500 times and 2000 times magnification were taken, respectively (see Figure 12).

Figure 12a shows the SEM image of PURD-3 under 500 times. It can be seen that PURD-3 has many macropores, the internal structure of the specimen is loose and not tight enough, and it can be clearly seen that there are many recycled aggregate particles on the surface of the specimen, which are bonded together through polyurethane. Part A1 in Figure 12a is enlarged to obtain Figure 12b. Figure 12b shows that a large amount of polyurethane adheres to the surface of RA, but the particle shape of RA can still be seen. The bonding between particles is not particularly tight. Meanwhile, the polyurethane exists in a linear form and plays a bridging role between RA particles, which is similar to the reinforcing effect of fiber in cement-based materials [51]. Figure 12c shows the SEM image of PURD-4 under 500 times. The image shows that there are some large pores and tiny cracks inside the specimen, but the pore size is reduced compared with the PURD-3, so the internal structure of the specimen is improved. Meanwhile, according to Figure 12d obtained by enlarging part A2 in Figure 12c, only some fine particles of RA can be seen on the surface of PURD-4, which is mainly because the RA particles are wrapped by a large amount of polyurethane, but due to the limited content of polyurethane, some fine particles of RA are exposed and adhered to the outside. It is more clearly seen that linear polyurethane has a strong adhesion effect, which can “pull” the various parts of the specimen together, providing a basis for the further connection of the specimen as a whole. Figure 12e is the SEM image time of the PURD-5 at 500 times. It is observed that the pores inside PURD-5 are smaller in size than those inside PURD-4, the internal structure is more compact, and the particle shape of the RA is also less visible. Due to the increase in the content of polyurethane, a large number of polyurethane-wrapped RA form polyurethane agglomerates, which make the integrity of the specimen more prominent [35,52]. Figure 12f is obtained by enlarging part A3 in Figure 12e. It can be seen from Figure 12f that a large amount of polyurethane wraps the RA to form an agglomerate structure, and a large number of polyurethane thick films appear, that is, the polyurethane covers and wraps the surface of the RA particles in the form of a film. Figure 12g is the SEM image of the PURD-6 at 500 times. It can be found that PURD-6 has only a few tiny pores, the overall structure of the specimen is quite complete, and the thin film structure of polyurethane can be clearly seen. The A4 part in Figure 12g is enlarged to obtain Figure 12h, from which it can be found that PURD-6 is covered with more thick polyurethane film than PURD-5, and almost no pores and cracks in the specimen can be seen. Moreover, the thickness of the polyurethane film also increases to a certain extent, which is mainly caused by the increase in the content of polyurethane.

In summary, the reinforcement effect of polymer can be attributed to the polymer film wrapping and binding the particles to create a spatially reticular membrane structure [27]. The above SEM microscopic image analysis shows that with the increase in WPU content, the internal pore and crack size of the PURD gradually decreases, and the number of both also decreases. Meanwhile, the polyurethane-wrapped RA particles gradually form agglomerates and finally produce a large number of polyurethane films. With the gradual increase in WPU content, the polyurethane film becomes increasingly thicker, which makes the bonding effect between the polyurethane and the RA particles become increasingly stronger, so the overall structure of the PURD becomes increasingly denser. This phenomenon can more fully explain the mechanical results that the shear strength of PURD increases with the increase in WPU content in the UU test. Due to the limitation of the SEM test magnification, the specific size of pores cannot be determined. In order to accurately determine the nanometer size of pores, a further transmission electron microscopy (TEM) test and specific surface area (BET) test are required.

## 5. Conclusions

To explore new environmentally friendly reinforcement materials and realize the recycling and utilization of construction waste, a new reinforcement method using PU to enhance RDW was proposed in this study, thus reducing the environmental pressure and resource shortage. The UU test and SEM test were conducted to investigate the reinforcement performance and mechanism of PU. The main conclusions are presented as follows:

(1) WPU can obviously improve the shear strength of RA. The shear strength of PURD increases with the increase in WPU content. When the WPU content is 6%, the shear strength reaches the maximum value, which is nearly 30% higher than that of PURD-5. However, the curing time has little effect on the reinforcement effect of WPU, and the increase in shear strength can be ignored. Therefore, WPU can directly exert all strength effects in a short curing time, and is relatively flexible in engineering application, which is a very effective and promising new type of high-molecular polymer reinforcement material.
(2) WPU has great influence on the ductility of RA. The failure strain of PURD increases with the increase in confining pressure, and first increases and then decreases with the increase in WPU content. The failure strain of PURD-5 reaches the maximum value. Compared with PURD-3, the failure strain of PURD-5 increases by 41%, 50%, 124%, and 99% at 7 days and 61%, 37%, 31%, and 41% at 28 days, respectively. The growth range is large, so PURD-5 shows the best ductility.
(3) WPU can obviously increase the cohesion of RA. The internal friction angle and cohesion of PURD increase with the increase in WPU content, which is highly consistent with the change law of shear strength. However, the increase in internal friction angle is small, only about 5%, and the increase in cohesion is obvious. At the curing time of 7 days, the cohesion of PURD-6 increases by 144%, 142%, and 91%, respectively, compared with those of PURD-3, PURD-4, and PURD-5. At the curing time of 28 days, the cohesion of PURD-6 increases by 141%, 134%, and 77%, respectively, compared with those of PURD-3, PURD-4, and PURD-5.
(4) The enhancement effect of the polymer can be attributed to the spatial reticular membrane structure produced by wrapping and bonding particles with the polymer film. With the increase in WPU content, the internal pore and crack size of PURD gradually decreases, and the number of them also decreases. Meanwhile, the polyurethane-wrapped RA particles gradually form agglomerates and finally produce a large number of polyurethane films. With the gradual increase in WPU content, the polyurethane film becomes increasingly thicker, which makes the bonding effect of the polyurethane on the RA particles become increasingly stronger, and the overall structure of the PURD becomes increasingly denser.
(5) In this test, the mechanical strength of RA is obtained on the basis that the mud content is 20.5%. If different practical projects need to obtain higher mechanical parameters, it is necessary to reduce the mud content of RA. Similarly, if the engineering has low requirements for mechanical parameters, the requirements for mud content can be appropriately relaxed. Thus, the processing steps for RA can be appropriately reduced.

## Figures and Tables

**Table 1:** Physical performance indexes of RA.

Physical Index	Natural Moisture Content (%)	Plasticity Index	Proportion (g/cm^3^)	Mud Content (%)	Apparent Density (kg/m^3^)
Index value	16	16.6	2.68	20.5	2.67

**Table 2:** Performance indexes of WPU solution.

Performance Index	Appearance	Solid Content (%)	pH Value (25 °C)	Viscosity (mPa∙S)	Film Forming Temperature (°C)
Index value	Milky white liquid	40	6~8	800~1500	20

**Table 3:** Test scheme for RA reinforced with different contents of WPU.

Specimen Number	Dry Density (g/cm^3^)	WPU Content (%)	Moisture Content (%)	Curing Time (Day)
PURD-3	1.6	3	16	7, 28
PURD-4		4		
PURD-5		5		
PURD-6		6		

## Data Availability

Not applicable.
